# The uncharted role of HER2 mutant alleles in breast cancer

**DOI:** 10.18632/oncotarget.28489

**Published:** 2023-10-31

**Authors:** Rashi Kalra, Bora Lim, Matthew J. Ellis, Shyam M. Kavuri

**Keywords:** metastasis, poziotinib, HER2, neratinib, invasive lobular breast carcinoma

## Abstract

Somatic HER2 mutations are a novel class of therapeutic targets across different cancer types. Treatment with the tyrosine kinase inhibitor (TKI) neratinib as a single agent continues to be evaluated in HER2-mutant metastatic disease. However, responses are heterogeneous, with frequent early progression. Herein, we discuss the under-explored effects of individual HER2 mutant alleles on therapeutic response, a role for HER2 mutation in metastatic propensity, and differences in patient outcomes in ER+ invasive lobular carcinoma (ILC) versus invasive ductal carcinoma (IDC). The preclinical efficacy of additional agents is also discussed, particularly the pan-HER inhibitor poziotinib.

## The clinical significance of HER2 activating somatic mutations in breast cancer

The Cancer Genome Atlas (TCGA) consortium first identified HER2 somatic mutations in non-HER2-amplified solid tumors over the last decade. Based on functional analyses and clinical trial data, our group and others have concluded that HER2 somatic mutations are indeed therapeutically targetable in estrogen receptor positive (ER+) non-HER2-amplified advanced breast cancer as well as other common solid tumors [1–12]. Importantly, HER2 mutation frequency varies across different tumor types. The prevalence is approximately 3% in primary ER+ breast cancer but increases to 6% in metastatic breast cancer (mBC), likely due to an association with acquired resistance to standard-of-care endocrine therapy [8, 13]. Furthermore, HER2 mutations are enriched in ER+ breast cancer with lobular histology, where the rates are reported to be between 5–26% [10, 14, 15]. Accumulated data in Phase 2 clinical trials of neratinib monotherapy in HER2 mutant cancers (NCT01670877 and NCT01953926) across multiple different cancer types show a modest response rate to neratinib as a single agent in ER+ mBC, but response durations are typically short [5, 7, 16, 17].

## The first-ever evidence of HER2 allele specific impact on patient outcome

We recently reported distinct prognostic effects of recurrent HER2 mutations in metastatic ER+ ILC versus IDC. HER2 L755 alterations (L755S or L755 insertions/deletions) are the commonest mutations in ILC, while HER2 exon insertions/deletions are more frequent in IDC. The METABRIC primary breast cancer sequencing data demonstrated that ER+ ILC patients harboring HER2 mutations have reduced overall survival in comparison to patients with HER2 mutant ER+ IDC. Of all the HER2 mutations evaluated, HER2 L755 alterations are selectively associated with poor outcome in lobular histology [18].

## The functional consequence of different HER2 alleles on therapeutic response and multi-organ metastasis in breast cancer

Based on these HER2 allele selective prognostic effects, the impact of different HER2 alleles on endocrine therapy sensitivity, modulation of signal transduction by neratinib, orthotopic tumor growth, and multi-organ metastasis was investigated. A key finding was that HER2 mutations constitutively activate HER2 oncogenic signaling more generally; mTOR signaling is particularly hyperactivated. Thus, mTOR activation is a likely focus for HER2 mutation-induced neratinib resistance [3, 18, 19].

By deploying several ER+ lobular *in vivo* models, it was evident that HER2 L755S induces resistance to neratinib and endocrine therapy in an estrogen-independent manner. In contrast, the recurrent HER2 extracellular domain mutation S310F remains highly estrogen-dependent and sensitive to endocrine therapy and neratinib. Additionally, HER2 L755S phenotype correlated with secondary ovary metastasis, an established clinical feature of lobular carcinoma, also in an estrogen-independent manner. Furthermore, these studies recapitulated the neratinib and endocrine resistance in two ER+ recurrent ductal HER2 mutant *in vivo* models, MCF7 HER2 L755S and HCI-003 PDX model (naturally harboring HER2^G778_P780 dup^) [18]. These findings summarized the neratinib and endocrine clinical resistance of HER2 recurrent mutant alleles in metastatic breast cancer, and underscored the pressing need for new treatment approaches beyond neratinib.

## Poziotinib inhibits HER2-mutant-driven endocrine or neratinib resistance and multi-organ metastasis

**Figure 1:**
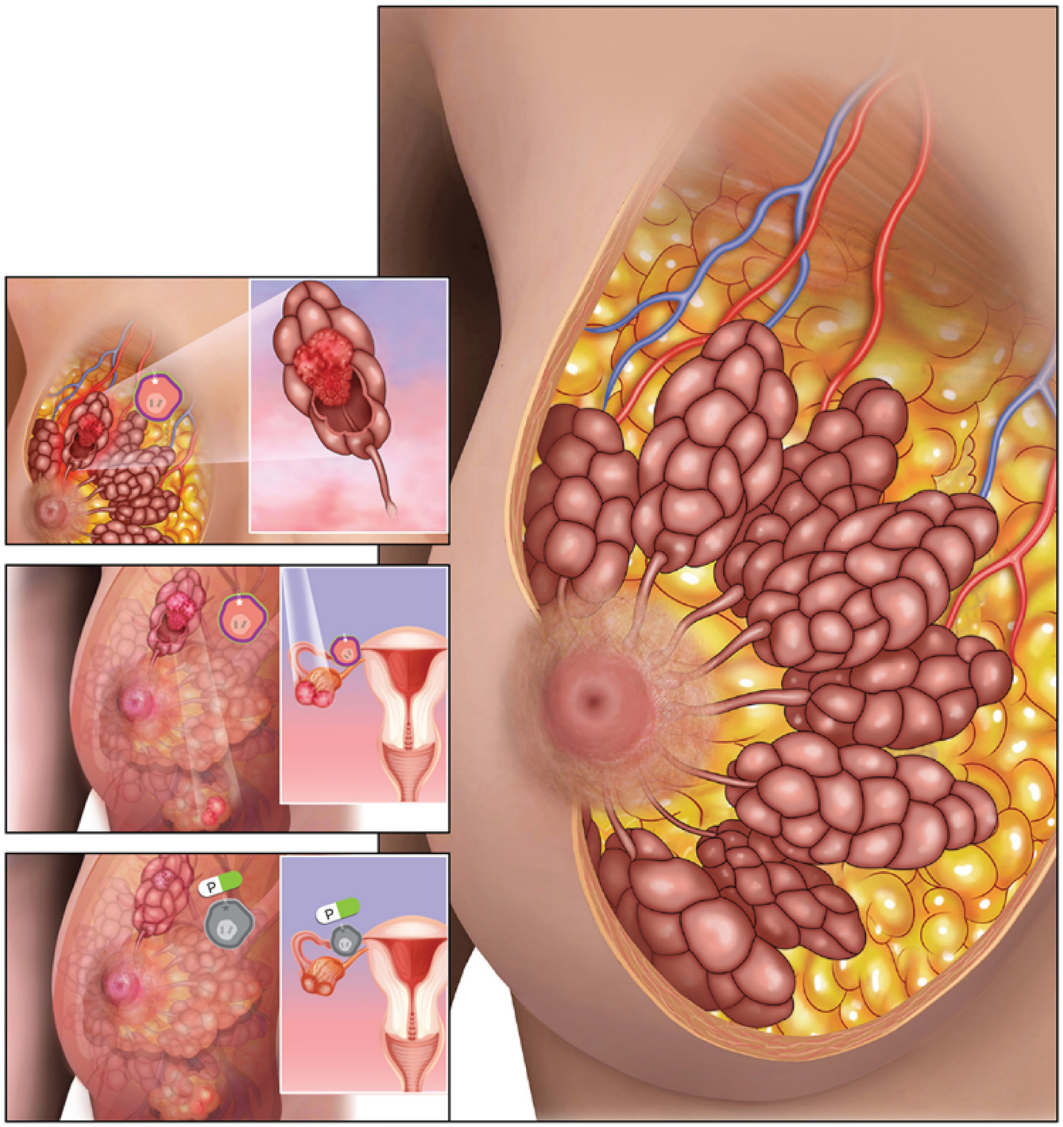
HER2 mutations are known therapeutic targets in ER+ HER2 non-amplified breast cancer. HER2 mutations are highly enriched in ER+ invasive lobular carcinoma (ILC) as compared to ER+ invasive ductal carcinoma (IDC). Further, HER2 mutations are associated with poor prognosis only in ER+ ILC. This figure depicts normal breast lobules (right; larger image), as well as HER2 tyrosine kinase domain mutation L755S (white *), a recurrent mutation in ER+ ILC that induces mammary tumor growth (left; first image), and metastasis to ovary (left; second image). The HER2 tyrosine kinase domain inhibitor poziotinib (white and green capsule) inhibits HER2 activation (left; third image (grey cells)) and thereby HER2-induced breast tumor growth and metastasis to ovary.

Using three independent HER2 mutant *in vivo* models, our study demonstrated that the pan-HER TKI poziotinib as a single agent inhibits HER2 mutant-driven PI3K-mTOR oncogenic signaling, endocrine resistance, efficacy in neratinib resistance settings, and multi-organ metastasis in breast cancer. Structural modeling studies revealed that poziotinib binds more closely to S755-mutant HER2 than to the HER2 wild type, suggesting that poziotinib has mutant-specific binding activity [18]. These findings may offer a possible explanation as to why poziotinib is more potent than neratinib in our HER2 mutant breast cancer preclinical models. These data thus suggest that poziotinib has broader therapeutic potential beyond metastatic Non-Small Cell Lung Cancer (NSCLC) [20, 21] with potential efficacy on HER2-positive breast cancer [22]. While poziotinib-associated clinical toxicity in NSCLC patients has been documented, treatment is feasible with individualized dose reductions [23]. The schematic representation of HER2 L755S-specific effects on therapeutic resistance and multi-organ metastasis in invasive lobular carcinoma is illustrated in Figure 1.

## Poziotinib preclinical therapeutic combinations

The existing evidence [21] suggests that poziotinib induces accumulation of HER2 on the cell surface and that the combination of poziotinib and the HER2 antibody-drug conjugate Trastuzumab-emtansine (TDM-1) potentiates antitumor activity in NSCLC preclinical models, suggesting that poziotinib in combination with TDM-1 is likely to be the best choice to achieve durable clinical response in the metastatic setting.

In summary, preclinical findings described above support clinical investigation of poziotinib in a subset of ER + mBC harboring HER2 somatic mutations and suggest further studies to evaluate poziotinib as a therapeutic agent in additional tumor types.

## Open questions and future directions

The preclinical and clinical actions of poziotinib have already been evaluated in patients with metastatic NSCLC carrying recurrent HER2 exon 20 insertions [20, 21]. Our research study has demonstrated that poziotinib is highly potent in neratinib-resistant HER2 L755S lobular and ductal breast cancer models, suggesting that poziotinib could be more effective than standard agents (endocrine and neratinib treatment) with relatively less clinical toxicity in HER2-missense metastatic breast cancer.

In current preclinical models and clinical trials, we are testing pan-HER TKI’s that are designed to inhibit HER2 WT activation in HER2 mutant cancers, but the pan HER TKI’s clinical responses are short lived with toxicity. Therefore, it is imperative to investigate HER2-mutant specific vulnerabilities and design HER2-mutant-selective treatment strategies for durable clinical responses with low levels of drug related toxicities. Recent data from the DESTINY-Lung phase II clinical trials have revealed the clinical benefit of Trastuzumab-Deruxtecan (T-DxD) in patients with HER2 mutant NSCLC, suggesting new therapeutic options for patients with HER2 mutant NSCLC cancers [24]. The future studies will test the HER2 allele specific clinical responses upon treatment with T-DxD in NSCLC and other solid tumors.

## References

[1] Ben-Baruch NE , et al. J Natl Compr Canc Netw. 2015; 13:1061–64. 10.6004/jnccn.2015.0131. 26358790 PMC4701428

[2] Bose R , et al. Cancer Discov. 2013; 3:224–37. 10.1158/2159-8290.CD-12-0349. 23220880 PMC3570596

[3] Croessmann S , et al. Clin Cancer Res. 2019; 25:277–89. 10.1158/1078-0432.CCR-18-1544. 30314968 PMC6320312

[4] Goncalves R , et al. Breast Cancer Res. 2014; 16:460. 25606588 10.1186/s13058-014-0460-4 PMC4384360

[5] Hyman DM , et al. Nature. 2018; 554:189–94. 10.1038/nature25475. 29420467 PMC5808581

[6] Kavuri SM , et al. Cancer Discov. 2015; 5:832–41. 10.1158/2159-8290.CD-14-1211. 26243863 PMC4527087

[7] Ma CX , et al. Clin Cancer Res. 2017; 23:5687–95. 10.1158/1078-0432.CCR-17-0900. 28679771 PMC6746403

[8] Nayar U , et al. Nat Genet. 2019; 51:207–16. 10.1038/s41588-018-0287-5. 30531871

[9] Razavi P , et al. Cancer Cell. 2018; 34:427–38.e6. 10.1016/j.ccell.2018.08.008. 30205045 PMC6327853

[10] Rosa-Rosa JM , et al. Cancers (Basel). 2019; 11:74. 10.3390/cancers11010074. 30641862 PMC6356653

[11] Weigelt B , et al. Cancer Discov. 2013; 3:145–47. 10.1158/2159-8290.CD-12-0585. 23400474

[12] Zabransky DJ , et al. Proc Natl Acad Sci U S A. 2015; 112:E6205–14. 10.1073/pnas.1516853112. 26508629 PMC4653184

[13] O’Leary B , et al. Cancer Discov. 2018; 8:1390–403. 10.1158/2159-8290.Cd-18-0264. 30206110 PMC6368247

[14] Desmedt C , et al. J Clin Oncol. 2016; 34:1872–81. 10.1200/JCO.2015.64.0334. 26926684

[15] Zhu S , et al. JCI Insight. 2018; 3:e97398. 10.1172/jci.insight.97398. 29669935 PMC5931118

[16] Smyth LM , et al. Cancer Discov. 2020; 10:198–213. 10.1158/2159-8290.CD-19-0966. 31806627 PMC7007377

[17] Ma CX , et al. Clin Cancer Res. 2022; 28:1258–67. 10.1158/1078-0432.CCR-21-3418. 35046057

[18] Kalra R , et al. Cancer Res. 2022; 82:2928–39. 10.1158/0008-5472.CAN-21-3106. 35736563 PMC9379360

[19] Sudhan DR , et al. Cancer Cell. 2020; 37:258–59. 10.1016/j.ccell.2020.01.010. 32049049 PMC7377274

[20] Robichaux JP , et al. Nat Med. 2018; 24:638–46. 10.1038/s41591-018-0007-9. 29686424 PMC5964608

[21] Robichaux JP , et al. Cancer Cell. 2019; 36:444–57.e7. 10.1016/j.ccell.2019.09.001. 31588020 PMC6944069

[22] Kim JY , et al. Int J Cancer. 2019; 145:1669–78. 10.1002/ijc.32188. 30720867

[23] Prelaj A , et al. Eur J Cancer. 2021; 149:235–48. 10.1016/j.ejca.2021.02.038. 33820681

[24] Li BT , et al. N Engl J Med. 2022; 386:1770–71. 10.1056/NEJMc2202305. 35507496 PMC11285070

